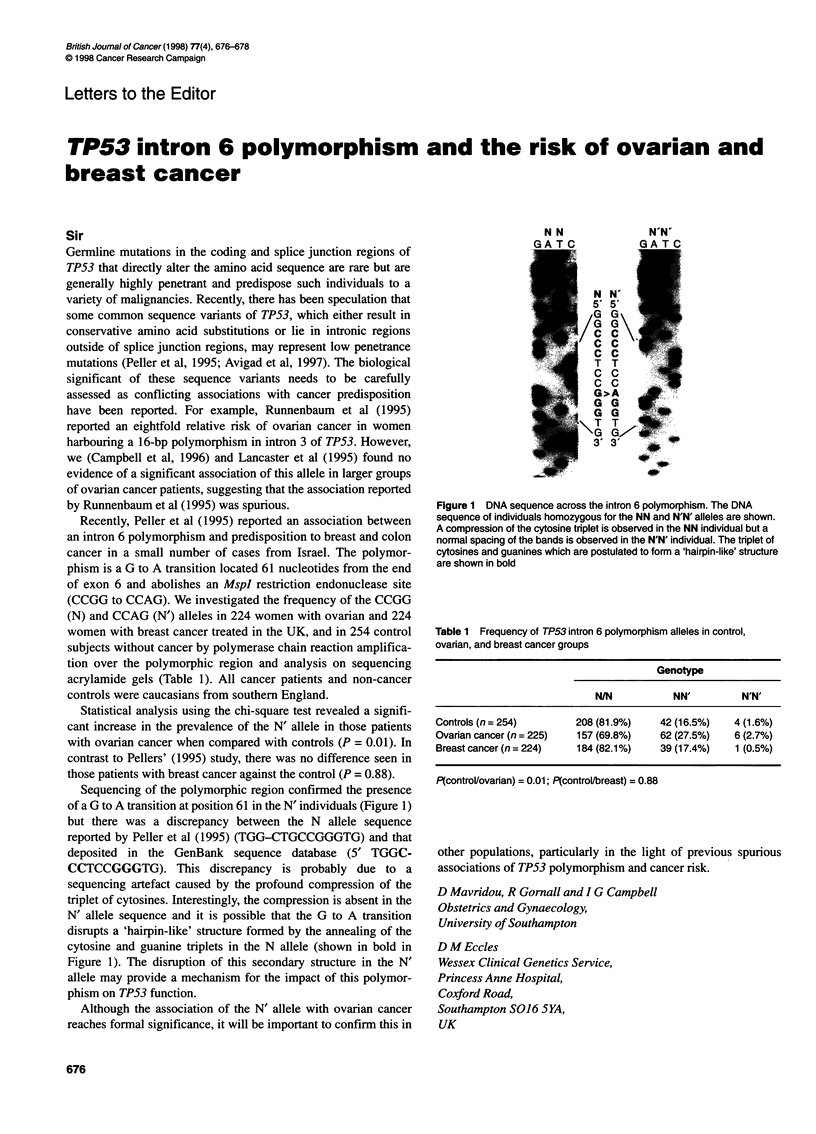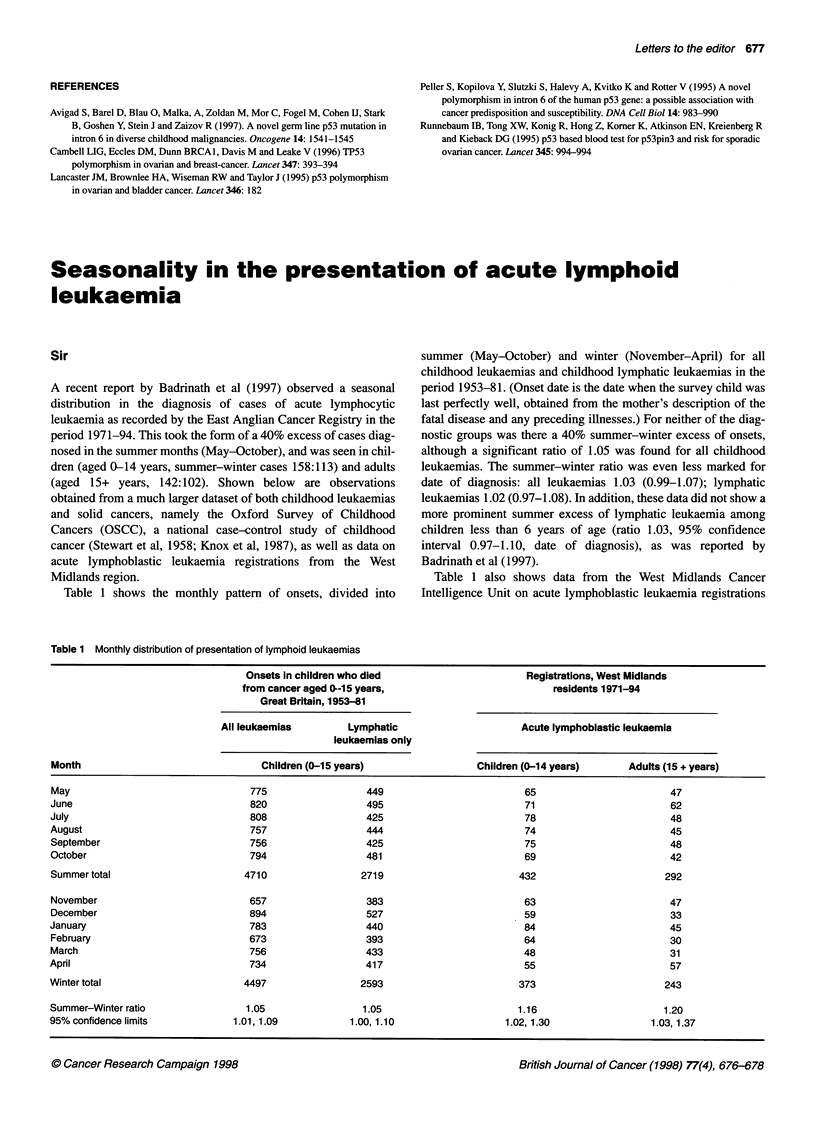# TP53 intron 6 polymorphism and the risk of ovarian and breast cancer.

**DOI:** 10.1038/bjc.1998.108

**Published:** 1998-02

**Authors:** D. Mavridou, R. Gornall, I. G. Campbell, D. M. Eccles

## Abstract

**Images:**


British Journal of Cancer (1998) 77(4), 676–678
© 1998 Cancer Research Campaign

Letters to the Editor

### *TP53* intron 6 polymorphism and the risk of ovarian and breast cancer

**Sir**

Germline mutations in the coding and splice junction regions of *TP53* that directly alter the amino acid sequence are rare but are generally highly penetrant and predispose such individuals to a variety of malignancies. Recently, there has been speculation that some common sequence variants of *TP53*, which either result in conservative amino acid substitutions or lie in intronic regions outside of splice junction regions, may represent low penetrance mutations (Peller et al, 1995; Avigad et al, 1997). The biological significant of these sequence variants needs to be carefully assessed as conflicting associations with cancer predisposition have been reported. For example, Runnenbaum et al (1995) reported an eightfold relative risk of ovarian cancer in women harbouring a 16-bp polymorphism in intron 3 of *TP53*. However, we (Campbell et al, 1996) and Lancaster et al (1995) found no evidence of a significant association of this allele in larger groups of ovarian cancer patients, suggesting that the association reported by Runnenbaum et al (1995) was spurious.

Recently, Peller et al (1995) reported an association between an intron 6 polymorphism and predisposition to breast and colon cancer in a small number of cases from Israel. The polymorphism is a G to A transition located 61 nucleotides from the end of exon 6 and abolishes an *MspI* restriction endonuclease site (CCGG to CCAG). We investigated the frequency of the CCGG (N) and CCAG (N′) alleles in 224 women with ovarian and 224 women with breast cancer treated in the UK, and in 254 control subjects without cancer by polymerase chain reaction amplification over the polymorphic region and analysis on sequencing acrylamide gels (Table 1). All cancer patients and non-cancer controls were caucasians from southern England.

Statistical analysis using the chi-square test revealed a significant increase in the prevalence of the N′ allele in those patients with ovarian cancer when compared with controls ($P = 0.01$). In contrast to Pellers' (1995) study, there was no difference seen in those patients with breast cancer against the control ($P = 0.88$).

Sequencing of the polymorphic region confirmed the presence of a G to A transition at position 61 in the N′ individuals (Figure 1) but there was a discrepancy between the N allele sequence reported by Peller et al (1995) (TGG–CTGCCGGGTG) and that deposited in the GenBank sequence database (5′ TGGC-CCTCCGGGTG). This discrepancy is probably due to a sequencing artefact caused by the profound compression of the triplet of cytosines. Interestingly, the compression is absent in the N′ allele sequence and it is possible that the G to A transition disrupts a 'hairpin-like' structure formed by the annealing of the cytosine and guanine triplets in the N allele (shown in bold in Figure 1). The disruption of this secondary structure in the N′ allele may provide a mechanism for the impact of this polymorphism on *TP53* function.

Although the association of the N′ allele with ovarian cancer reaches formal significance, it will be important to confirm this in other populations, particularly in the light of previous spurious associations of *TP53* polymorphism and cancer risk.

**Figure 1** DNA sequence across the intron 6 polymorphism. The DNA sequence of individuals homozygous for the **NN** and **N′N′** alleles are shown. A compression of the cytosine triplet is observed in the **NN** individual but a normal spacing of the bands is observed in the **N′N′** individual. The triplet of cytosines and guanines which are postulated to form a 'hairpin-like' structure are shown in bold

**Table 1** Frequency of *TP53* intron 6 polymorphism alleles in control, ovarian, and breast cancer groups

| | Genotype | | |
|---|---|---|---|
| | **N/N** | **NN′** | **N′N′** |
| Controls ($n$ = 254) | 208 (81.9%) | 42 (16.5%) | 4 (1.6%) |
| Ovarian cancer ($n$ = 225) | 157 (69.8%) | 62 (27.5%) | 6 (2.7%) |
| Breast cancer ($n$ = 224) | 184 (82.1%) | 39 (17.4%) | 1 (0.5%) |

$P$(control/ovarian) = 0.01; $P$(control/breast) = 0.88

*D Mavridou, R Gornall and I G Campbell*
*Obstetrics and Gynaecology,*
*University of Southampton*

*D M Eccles*
*Wessex Clinical Genetics Service,*
*Princess Anne Hospital,*
*Coxford Road,*
*Southampton SO16 5YA,*
*UK*

**REFERENCES**

Avigad S, Barel D, Blau O, Malka, A, Zoldan M, Mor C, Fogel M, Cohen IJ, Stark B, Goshen Y, Stein J and Zaizov R (1997). A novel germ line p53 mutation in intron 6 in diverse childhood malignancies. *Oncogene* **14**: 1541–1545

Cambell LIG, Eccles DM, Dunn BRCA1, Davis M and Leake V (1996) TP53 polymorphism in ovarian and breast-cancer. *Lancet* **347**: 393–394

Lancaster JM, Brownlee HA, Wiseman RW and Taylor J (1995) p53 polymorphism in ovarian and bladder cancer. *Lancet* **346**: 182

Peller S, Kopilova Y, Slutzki S, Halevy A, Kvitko K and Rotter V (1995) A novel polymorphism in intron 6 of the human p53 gene: a possible association with cancer predisposition and susceptibility. *DNA Cell Biol* **14**: 983–990

Runnebaum IB, Tong XW, Konig R, Hong Z, Korner K, Atkinson EN, Kreienberg R and Kieback DG (1995) p53 based blood test for p53pin3 and risk for sporadic ovarian cancer. *Lancet* **345**: 994–994